# Supplementary material for: Analysis of PPARγ Signaling Activity in Psoriasis
Source: Int J Mol Sci. 2021 Aug 10;22(16):8603. doi: 10.3390/ijms22168603 (PMC8395241; doi:10.3390/ijms22168603)
Supplement: Supplementary file 1 [file ijms-22-08603-s001.zip › Supplemental materials_Analysis of PPARg signaling activity in psoriasis/Pathway models/Models images and html files/PPARG inhibits phenotypes assotiated with psoriasis/124667.html]

fibrosis


# Disease fibrosis

|  |  |
| --- | --- |
| URN | urn:agi-meshdis:Fibrosis |
| Total Entities | 32 |
| Connectivity | 9332 |
| Name | fibrosis |

---

|  |  |
| --- | --- |
| ChildConcepts | scar |
|  | pulmonary fibrosis |
|  | fibromyalgia |
|  | cystic fibrosis |
|  | nephrogenic systemic fibrosis |
|  | radiation fibrosis |
|  | peritoneal fibrosis |
|  | biliary tract fibrosis |
|  | breast fibrosis |
|  | injection site fibrosis |
|  | intestinal fibrosis |
|  | myelofibrosis |
|  | muscle fibrosis |
|  | ocular fibrosis |
|  | penile fibrosis |
|  | mediastinal fibrosis |
|  | eosinophilic angiocentric fibrosis |
|  | ureteral fibrosis |
|  | vascular fibrosis |
|  | myocardial fibrosis |
|  | pleural fibrosis |
|  | pancreatic fibrosis |
|  | interstitial fibrosis |
|  | skin fibrosis |
|  | oral submucous fibrosis |
|  | retroperitoneal fibrosis |
|  | experimental fibrosis |
|  | liver fibrosis |
|  | cholangiofibrosis |
|  | perisinusoidal fibrosis |
|  | cardiosclerosis |
|  | congenital fibrosis of extraocular muscles type 1 |

---

|  |  |
| --- | --- |
| Pathway | Complement Cascade Activation by Pentraxins |
|  | Cardiomyocyte Dysfunction |
|  | Dilated Cardiomyopathy |
|  | Cardiomyocyte Hypertrophy |
|  | Hypertrophic Cardiomyopathy |
|  | Smooth Muscle Cell Dysfunction in Pulmonary Hypertension |
|  | Myocardial Remodeling |
|  | Tubular Cell Dysfunction in Progressive Diabetic Nephropathy |
|  | Mesangial Cell Dysfunction in Diabetic Nephropathy |
|  | Hepatic Stellate Cells in non-Alcoholic Fatty Liver Disease |
|  | Graves Ophthalmopathy |
|  | Hodgkin and Reed-Sternberg Cells in Hodgkin Lymphoma |
|  | Hodgkin Lymphoma |
|  | Periostin (POSTN) Production by Airway Epithelium in Asthma |
|  | IL17F Signaling in Bronchial Epithelial Cell in Asthma |
|  | B-Cells Function in Systemic Scleroderma |
|  | Skin Fibrosis |
|  | Ca2+ Overload in Muscular Dystrophies |
|  | Muscular Dystrophies |
|  | Podocyte Dysfunction in Glomerulonephritis |
|  | Mesangial Cell Dysfunction in Glomerulonephritis |
|  | Interstitial Fibroblasts in Pyelonephritis |
|  | Endothelial Cell Dysfunction in Pyelonephritis |
|  | Ethanol Induced Hepatotoxicity |
|  | PPARG inhibits phenotypes assotiated with psoriasis |
|  | efects selected |
|  | Neighbors of fibrosis |
|  | 3\_The role of interstitial fibroblasts in pyelonephritis |
|  | 3\_Mesangial cell dysfunction in glomerulonephritis |
|  | 2\_Cardiomyocyte hypertrophy |
|  | 3\_Cardiomyocyte dysfunction unrelated to cell junctions in ARVD |
|  | 2\_The intense proliferation and contractile activity of smooth muscle cells in pulmonary hypertension |
|  | 4\_2\_Airway smooth muscle cells proliferation and airway tissue remodeling |

---

|  |  |
| --- | --- |
| MedScan ID | 9018567 |

---

|  |  |
| --- | --- |
| Alias | portal fibrosis |
|  | Fibrous Replacement |
|  | Fibrosis |
|  | Fibrous Thickening |
|  | Fibroses |
|  | portal fibroses |
|  | Fibrous Thickenings |
|  | Desmoplasia |
|  | Desmoplasias |
|  | Fibrous Repair |
|  | myxofibrosis |
|  | Fibroplasias |
|  | Fibroplasia |
|  | cirrhotic |

---

|  |  |
| --- | --- |
| MeSH ID | D005355 |
|  | M0008453 |

---

|  |  |
| --- | --- |
| MeSH Heading | Fibrosis |

---
